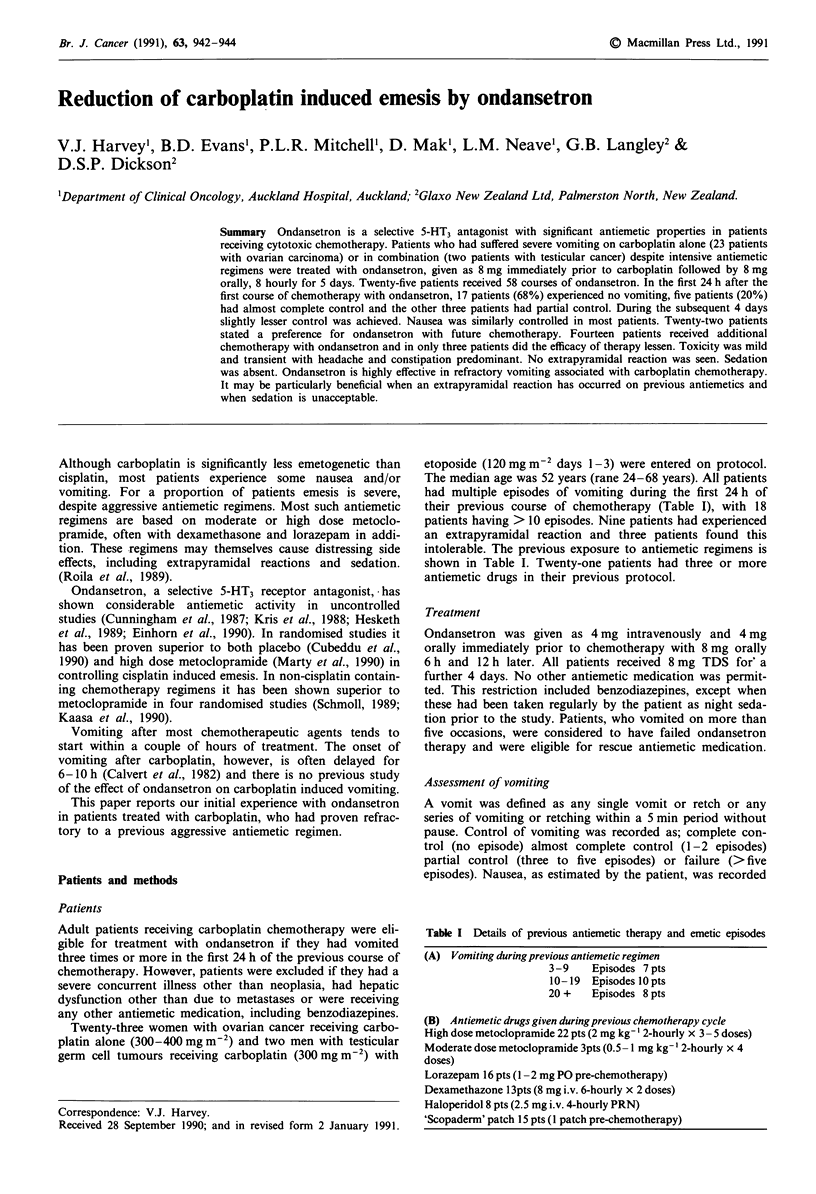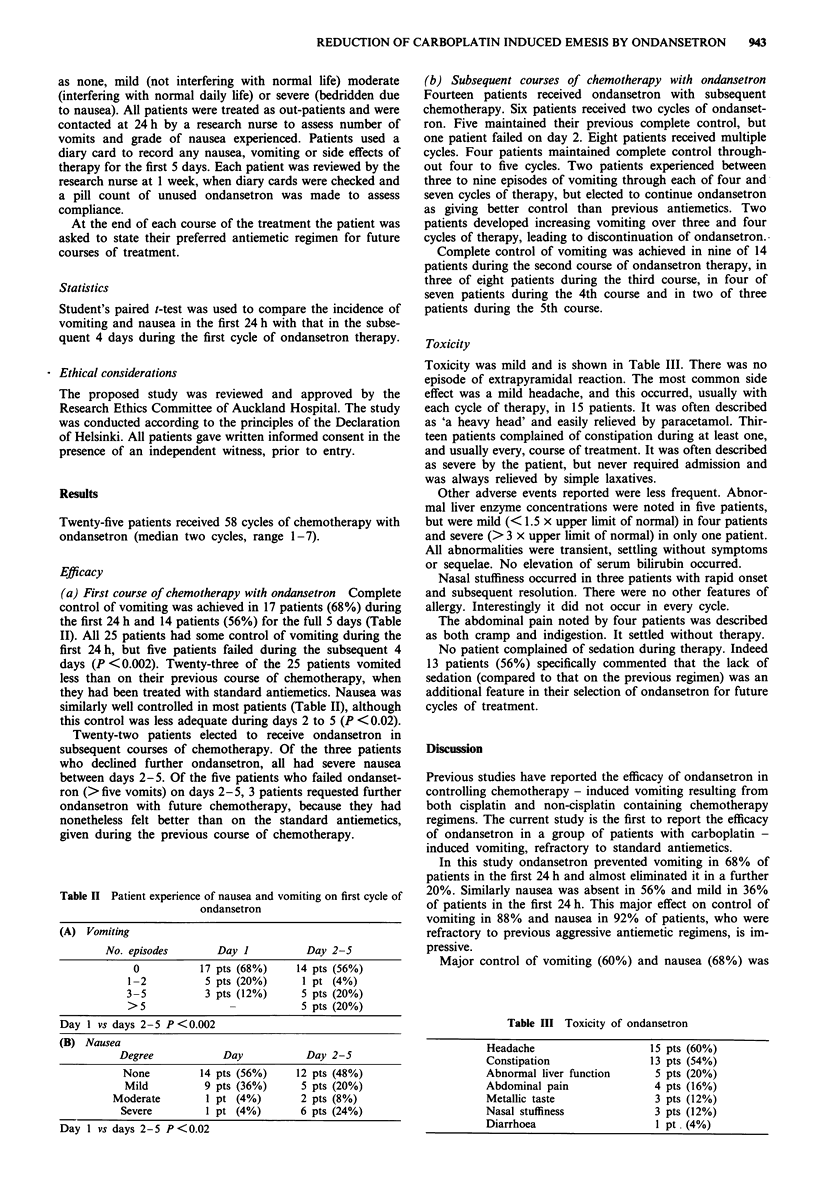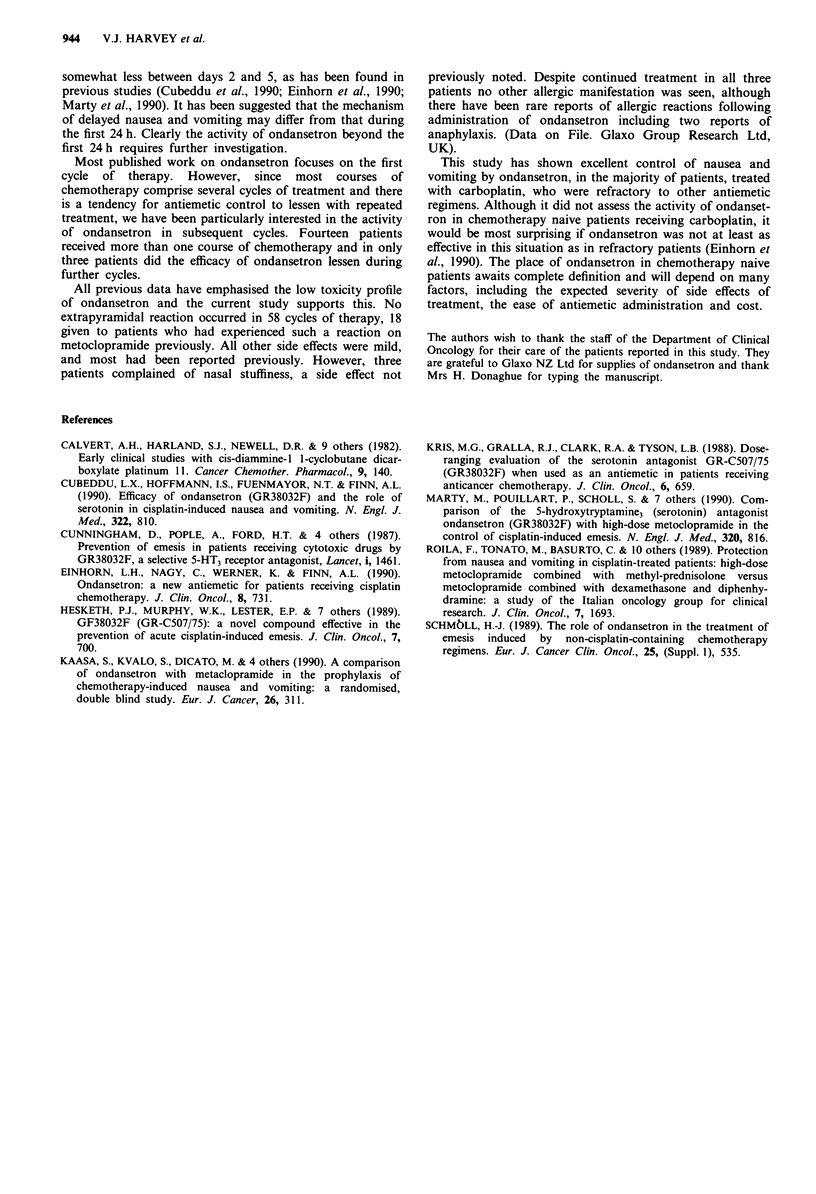# Reduction of carboplatin induced emesis by ondansetron.

**DOI:** 10.1038/bjc.1991.206

**Published:** 1991-06

**Authors:** V. J. Harvey, B. D. Evans, P. L. Mitchell, D. Mak, L. M. Neave, G. B. Langley, D. S. Dickson

**Affiliations:** Department of Clinical Oncology, Auckland Hospital, New Zealand.

## Abstract

Ondansetron is a selective 5-HT3 antagonist with significant antiemetic properties in patients receiving cytotoxic chemotherapy. Patients who had suffered severe vomiting on carboplatin alone (23 patients with ovarian carcinoma) or in combination (two patients with testicular cancer) despite intensive antiemetic regimens were treated with ondansetron, given as 8 mg immediately prior to carboplatin followed by 8 mg orally, 8 hourly for 5 days. Twenty-five patients received 58 courses of ondansetron. In the first 24 h after the first course of chemotherapy with ondansetron, 17 patients (68%) experienced no vomiting, five patients (20%) had almost complete control and the other three patients had partial control. During the subsequent 4 days slightly lesser control was achieved. Nausea was similarly controlled in most patients. Twenty-two patients stated a preference for ondansetron with future chemotherapy. Fourteen patients received additional chemotherapy with ondansetron and in only three patients did the efficacy of therapy lessen. Toxicity was mild and transient with headache and constipation predominant. No extrapyramidal reaction was seen. Sedation was absent. Ondansetron is highly effective in refractory vomiting associated with carboplatin chemotherapy. It may be particularly beneficial when an extrapyramidal reaction has occurred on previous antiemetics and when sedation is unacceptable.


Br. J. Cancer (1991), 63, 942-944 © Macmillan Press Ltd., 1991

### Reduction of carboplatin induced emesis by ondansetron

V.J. Harvey[1], B.D. Evans[1], P.L.R. Mitchell[1], D. Mak[1], L.M. Neave[1], G.B. Langley[2] & D.S.P. Dickson[2]

[1]*Department of Clinical Oncology, Auckland Hospital, Auckland;* [2]*Glaxo New Zealand Ltd, Palmerston North, New Zealand.*

**Summary** Ondansetron is a selective 5-$HT_3$ antagonist with significant antiemetic properties in patients receiving cytotoxic chemotherapy. Patients who had suffered severe vomiting on carboplatin alone (23 patients with ovarian carcinoma) or in combination (two patients with testicular cancer) despite intensive antiemetic regimens were treated with ondansetron, given as 8 mg immediately prior to carboplatin followed by 8 mg orally, 8 hourly for 5 days. Twenty-five patients received 58 courses of ondansetron. In the first 24 h after the first course of chemotherapy with ondansetron, 17 patients (68%) experienced no vomiting, five patients (20%) had almost complete control and the other three patients had partial control. During the subsequent 4 days slightly lesser control was achieved. Nausea was similarly controlled in most patients. Twenty-two patients stated a preference for ondansetron with future chemotherapy. Fourteen patients received additional chemotherapy with ondansetron and in only three patients did the efficacy of therapy lessen. Toxicity was mild and transient with headache and constipation predominant. No extrapyramidal reaction was seen. Sedation was absent. Ondansetron is highly effective in refractory vomiting associated with carboplatin chemotherapy. It may be particularly beneficial when an extrapyramidal reaction has occurred on previous antiemetics and when sedation is unacceptable.

Although carboplatin is significantly less emetogenetic than cisplatin, most patients experience some nausea and/or vomiting. For a proportion of patients emesis is severe, despite aggressive antiemetic regimens. Most such antiemetic regimens are based on moderate or high dose metoclopramide, often with dexamethasone and lorazepam in addition. These regimens may themselves cause distressing side effects, including extrapyramidal reactions and sedation. (Roila *et al.*, 1989).

Ondansetron, a selective 5-$HT_3$ receptor antagonist, has shown considerable antiemetic activity in uncontrolled studies (Cunningham *et al.*, 1987; Kris *et al.*, 1988; Hesketh *et al.*, 1989; Einhorn *et al.*, 1990). In randomised studies it has been proven superior to both placebo (Cubeddu *et al.*, 1990) and high dose metoclopramide (Marty *et al.*, 1990) in controlling cisplatin induced emesis. In non-cisplatin containing chemotherapy regimens it has been shown superior to metoclopramide in four randomised studies (Schmoll, 1989; Kaasa *et al.*, 1990).

Vomiting after most chemotherapeutic agents tends to start within a couple of hours of treatment. The onset of vomiting after carboplatin, however, is often delayed for 6-10 h (Calvert *et al.*, 1982) and there is no previous study of the effect of ondansetron on carboplatin induced vomiting.

This paper reports our initial experience with ondansetron in patients treated with carboplatin, who had proven refractory to a previous aggressive antiemetic regimen.

### Patients and methods

### *Patients*

Adult patients receiving carboplatin chemotherapy were eligible for treatment with ondansetron if they had vomited three times or more in the first 24 h of the previous course of chemotherapy. However, patients were excluded if they had a severe concurrent illness other than neoplasia, had hepatic dysfunction other than due to metastases or were receiving any other antiemetic medication, including benzodiazepines.

Twenty-three women with ovarian cancer receiving carboplatin alone (300-400 mg $m^{-2}$) and two men with testicular germ cell tumours receiving carboplatin (300 mg $m^{-2}$) with etoposide (120 mg $m^{-2}$ days 1-3) were entered on protocol. The median age was 52 years (rane 24-68 years). All patients had multiple episodes of vomiting during the first 24 h of their previous course of chemotherapy (Table I), with 18 patients having > 10 episodes. Nine patients had experienced an extrapyramidal reaction and three patients found this intolerable. The previous exposure to antiemetic regimens is shown in Table I. Twenty-one patients had three or more antiemetic drugs in their previous protocol.

### *Treatment*

Ondansetron was given as 4 mg intravenously and 4 mg orally immediately prior to chemotherapy with 8 mg orally 6 h and 12 h later. All patients received 8 mg TDS for a further 4 days. No other antiemetic medication was permitted. This restriction included benzodiazepines, except when these had been taken regularly by the patient as night sedation prior to the study. Patients, who vomited on more than five occasions, were considered to have failed ondansetron therapy and were eligible for rescue antiemetic medication.

### *Assessment of vomiting*

A vomit was defined as any single vomit or retch or any series of vomiting or retching within a 5 min period without pause. Control of vomiting was recorded as; complete control (no episode) almost complete control (1-2 episodes) partial control (three to five episodes) or failure (>five episodes). Nausea, as estimated by the patient, was recorded

**Table I** Details of previous antiemetic therapy and emetic episodes

| | | |
|---|---|---|
| **(A)** *Vomiting during previous antiemetic regimen* | | |
| 3-9 | Episodes | 7 pts |
| 10-19 | Episodes | 10 pts |
| 20 + | Episodes | 8 pts |
| **(B)** *Antiemetic drugs given during previous chemotherapy cycle* | | |
| High dose metoclopramide 22 pts (2 mg $kg^{-1}$ 2-hourly × 3-5 doses) | | |
| Moderate dose metoclopramide 3pts (0.5-1 mg $kg^{-1}$ 2-hourly × 4 doses) | | |
| Lorazepam 16 pts (1-2 mg PO pre-chemotherapy) | | |
| Dexamethazone 13pts (8 mg i.v. 6-hourly × 2 doses) | | |
| Haloperidol 8 pts (2.5 mg i.v. 4-hourly PRN) | | |
| 'Scopaderm' patch 15 pts (1 patch pre-chemotherapy) | | |

Correspondence: V.J. Harvey.
Received 28 September 1990; and in revised form 2 January 1991.

as none, mild (not interfering with normal life) moderate (interfering with normal daily life) or severe (bedridden due to nausea). All patients were treated as out-patients and were contacted at 24 h by a research nurse to assess number of vomits and grade of nausea experienced. Patients used a diary card to record any nausea, vomiting or side effects of therapy for the first 5 days. Each patient was reviewed by the research nurse at 1 week, when diary cards were checked and a pill count of unused ondansetron was made to assess compliance.

At the end of each course of the treatment the patient was asked to state their preferred antiemetic regimen for future courses of treatment.

*Statistics*

Student's paired $t$-test was used to compare the incidence of vomiting and nausea in the first 24 h with that in the subsequent 4 days during the first cycle of ondansetron therapy.

*Ethical considerations*

The proposed study was reviewed and approved by the Research Ethics Committee of Auckland Hospital. The study was conducted according to the principles of the Declaration of Helsinki. All patients gave written informed consent in the presence of an independent witness, prior to entry.

### Results

Twenty-five patients received 58 cycles of chemotherapy with ondansetron (median two cycles, range 1–7).

*Efficacy*

*(a) First course of chemotherapy with ondansetron* Complete control of vomiting was achieved in 17 patients (68%) during the first 24 h and 14 patients (56%) for the full 5 days (Table II). All 25 patients had some control of vomiting during the first 24 h, but five patients failed during the subsequent 4 days ($P < 0.002$). Twenty-three of the 25 patients vomited less than on their previous course of chemotherapy, when they had been treated with standard antiemetics. Nausea was similarly well controlled in most patients (Table II), although this control was less adequate during days 2 to 5 ($P < 0.02$).

Twenty-two patients elected to receive ondansetron in subsequent courses of chemotherapy. Of the three patients who declined further ondansetron, all had severe nausea between days 2–5. Of the five patients who failed ondansetron (> five vomits) on days 2–5, 3 patients requested further ondansetron with future chemotherapy, because they had nonetheless felt better than on the standard antiemetics, given during the previous course of chemotherapy.

**Table II** Patient experience of nausea and vomiting on first cycle of ondansetron

**(A)** *Vomiting*

| *No. episodes* | *Day 1* | *Day 2–5* |
|---|---|---|
| 0 | 17 pts (68%) | 14 pts (56%) |
| 1–2 | 5 pts (20%) | 1 pt (4%) |
| 3–5 | 3 pts (12%) | 5 pts (20%) |
| >5 | – | 5 pts (20%) |

Day 1 *vs* days 2–5 $P < 0.002$

**(B)** *Nausea*

| *Degree* | *Day* | *Day 2–5* |
|---|---|---|
| None | 14 pts (56%) | 12 pts (48%) |
| Mild | 9 pts (36%) | 5 pts (20%) |
| Moderate | 1 pt (4%) | 2 pts (8%) |
| Severe | 1 pt (4%) | 6 pts (24%) |

Day 1 *vs* days 2–5 $P < 0.02$

*(b) Subsequent courses of chemotherapy with ondansetron* Fourteen patients received ondansetron with subsequent chemotherapy. Six patients received two cycles of ondansetron. Five maintained their previous complete control, but one patient failed on day 2. Eight patients received multiple cycles. Four patients maintained complete control throughout four to five cycles. Two patients experienced between three to nine episodes of vomiting through each of four and seven cycles of therapy, but elected to continue ondansetron as giving better control than previous antiemetics. Two patients developed increasing vomiting over three and four cycles of therapy, leading to discontinuation of ondansetron.

Complete control of vomiting was achieved in nine of 14 patients during the second course of ondansetron therapy, in three of eight patients during the third course, in four of seven patients during the 4th course and in two of three patients during the 5th course.

*Toxicity*

Toxicity was mild and is shown in Table III. There was no episode of extrapyramidal reaction. The most common side effect was a mild headache, and this occurred, usually with each cycle of therapy, in 15 patients. It was often described as 'a heavy head' and easily relieved by paracetamol. Thirteen patients complained of constipation during at least one, and usually every, course of treatment. It was often described as severe by the patient, but never required admission and was always relieved by simple laxatives.

Other adverse events reported were less frequent. Abnormal liver enzyme concentrations were noted in five patients, but were mild (< 1.5 × upper limit of normal) in four patients and severe (> 3 × upper limit of normal) in only one patient. All abnormalities were transient, settling without symptoms or sequelae. No elevation of serum bilirubin occurred.

Nasal stuffiness occurred in three patients with rapid onset and subsequent resolution. There were no other features of allergy. Interestingly it did not occur in every cycle.

The abdominal pain noted by four patients was described as both cramp and indigestion. It settled without therapy.

No patient complained of sedation during therapy. Indeed 13 patients (56%) specifically commented that the lack of sedation (compared to that on the previous regimen) was an additional feature in their selection of ondansetron for future cycles of treatment.

### Discussion

Previous studies have reported the efficacy of ondansetron in controlling chemotherapy – induced vomiting resulting from both cisplatin and non-cisplatin containing chemotherapy regimens. The current study is the first to report the efficacy of ondansetron in a group of patients with carboplatin – induced vomiting, refractory to standard antiemetics.

In this study ondansetron prevented vomiting in 68% of patients in the first 24 h and almost eliminated it in a further 20%. Similarly nausea was absent in 56% and mild in 36% of patients in the first 24 h. This major effect on control of vomiting in 88% and nausea in 92% of patients, who were refractory to previous aggressive antiemetic regimens, is impressive.

Major control of vomiting (60%) and nausea (68%) was

**Table III** Toxicity of ondansetron

| | |
|---|---|
| Headache | 15 pts (60%) |
| Constipation | 13 pts (54%) |
| Abnormal liver function | 5 pts (20%) |
| Abdominal pain | 4 pts (16%) |
| Metallic taste | 3 pts (12%) |
| Nasal stuffiness | 3 pts (12%) |
| Diarrhoea | 1 pt (4%) |

somewhat less between days 2 and 5, as has been found in previous studies (Cubeddu *et al.*, 1990; Einhorn *et al.*, 1990; Marty *et al.*, 1990). It has been suggested that the mechanism of delayed nausea and vomiting may differ from that during the first 24 h. Clearly the activity of ondansetron beyond the first 24 h requires further investigation.

Most published work on ondansetron focuses on the first cycle of therapy. However, since most courses of chemotherapy comprise several cycles of treatment and there is a tendency for antiemetic control to lessen with repeated treatment, we have been particularly interested in the activity of ondansetron in subsequent cycles. Fourteen patients received more than one course of chemotherapy and in only three patients did the efficacy of ondansetron lessen during further cycles.

All previous data have emphasised the low toxicity profile of ondansetron and the current study supports this. No extrapyramidal reaction occurred in 58 cycles of therapy, 18 given to patients who had experienced such a reaction on metoclopramide previously. All other side effects were mild, and most had been reported previously. However, three patients complained of nasal stuffiness, a side effect not previously noted. Despite continued treatment in all three patients no other allergic manifestation was seen, although there have been rare reports of allergic reactions following administration of ondansetron including two reports of anaphylaxis. (Data on File. Glaxo Group Research Ltd, UK).

This study has shown excellent control of nausea and vomiting by ondansetron, in the majority of patients, treated with carboplatin, who were refractory to other antiemetic regimens. Although it did not assess the activity of ondansetron in chemotherapy naive patients receiving carboplatin, it would be most surprising if ondansetron was not at least as effective in this situation as in refractory patients (Einhorn *et al.*, 1990). The place of ondansetron in chemotherapy naive patients awaits complete definition and will depend on many factors, including the expected severity of side effects of treatment, the ease of antiemetic administration and cost.

The authors wish to thank the staff of the Department of Clinical Oncology for their care of the patients reported in this study. They are grateful to Glaxo NZ Ltd for supplies of ondansetron and thank Mrs H. Donaghue for typing the manuscript.

### References

CALVERT, A.H., HARLAND, S.J., NEWELL, D.R. & 9 others (1982). Early clinical studies with cis-diammine-1 1-cyclobutane dicarboxylate platinum 11. *Cancer Chemother. Pharmacol.*, **9**, 140.

CUBEDDU, L.X., HOFFMANN, I.S., FUENMAYOR, N.T. & FINN, A.L. (1990). Efficacy of ondansetron (GR38032F) and the role of serotonin in cisplatin-induced nausea and vomiting. *N. Engl. J. Med.*, **322**, 810.

CUNNINGHAM, D., POPLE, A., FORD, H.T. & 4 others (1987). Prevention of emesis in patients receiving cytotoxic drugs by GR38032F, a selective 5-$HT_3$ receptor antagonist, *Lancet*, **i**, 1461.

EINHORN, L.H., NAGY, C., WERNER, K. & FINN, A.L. (1990). Ondansetron: a new antiemetic for patients receiving cisplatin chemotherapy. *J. Clin. Oncol.*, **8**, 731.

HESKETH, P.J., MURPHY, W.K., LESTER, E.P. & 7 others (1989). GF38032F (GR-C507/75): a novel compound effective in the prevention of acute cisplatin-induced emesis. *J. Clin. Oncol.*, **7**, 700.

KAASA, S., KVALO, S., DICATO, M. & 4 others (1990). A comparison of ondansetron with metaclopramide in the prophylaxis of chemotherapy-induced nausea and vomiting: a randomised, double blind study. *Eur. J. Cancer*, **26**, 311.

KRIS, M.G., GRALLA, R.J., CLARK, R.A. & TYSON, L.B. (1988). Dose-ranging evaluation of the serotonin antagonist GR-C507/75 (GR38032F) when used as an antiemetic in patients receiving anticancer chemotherapy. *J. Clin. Oncol.*, **6**, 659.

MARTY, M., POUILLART, P., SCHOLL, S. & 7 others (1990). Comparison of the 5-hydroxytryptamine$_3$ (serotonin) antagonist ondansetron (GR38032F) with high-dose metoclopramide in the control of cisplatin-induced emesis. *N. Engl. J. Med.*, **320**, 816.

ROILA, F., TONATO, M., BASURTO, C. & 10 others (1989). Protection from nausea and vomiting in cisplatin-treated patients: high-dose metoclopramide combined with methyl-prednisolone versus metoclopramide combined with dexamethasone and diphenhydramine: a study of the Italian oncology group for clinical research. *J. Clin. Oncol.*, **7**, 1693.

SCHMÖLL, H.-J. (1989). The role of ondansetron in the treatment of emesis induced by non-cisplatin-containing chemotherapy regimens. *Eur. J. Cancer Clin. Oncol.*, **25**, (Suppl. 1), 535.